# Serum Uric Acid Levels and Bone Mineral Density in Peri- and Postmenopausal Korean Women: A Cross-sectional Study on 3,566 Cases

**DOI:** 10.34172/aim.34066

**Published:** 2025-06-01

**Authors:** Ki-Bong Park, Minsu Han, Su Jin Choi, Gyung-Min Park, Young-Jee Jeon, Doo-Ho Lim

**Affiliations:** ^1^Department of Orthopedic Surgery, Ulsan University Hospital, University of Ulsan College of Medicine, Ulsan, South Korea; ^2^Department of Internal Medicine, Ulsan University Hospital, University of Ulsan College of Medicine, Ulsan, South Korea; ^3^Department of Family Medicine, Ulsan University Hospital, University of Ulsan College of Medicine, Ulsan, South Korea

**Keywords:** Bone mineral density, Menopause, Osteoporosis, Uric acid

## Abstract

**Background:**

Uric acid (UA) may influence bone health through its antioxidant and pro-oxidant properties. While previous studies have investigated the relationship between serum UA levels and bone mineral density (BMD), their findings have been conflicting. This study aimed to examine the impact of serum UA levels on BMD in peri- and postmenopausal Korean women.

**Methods:**

We evaluated 3,566 women aged 50–80 years who voluntarily underwent laboratory tests and BMD measurements as part of a general health examination between March 2014 and March 2020. Participants were stratified into quartiles according to their serum UA levels. Univariate and multivariate analyses were performed to assess the association between serum UA levels and BMD.

**Results:**

The mean age of the participants was 56.9±5.8 years. BMD at the lumbar spine and hip was significantly higher in women with elevated serum UA levels, showing a continuous increase across the quartiles. Furthermore, after adjusting for covariates, the mean total lumbar spine BMD increased from 0.892 g/cm^2^ (95% CI: 0.884–0.900) in the lowest UA quartile to 0.918 g/cm^2^ (95% CI: 0.909–0.927) in the highest quartile (*P*<0.001). Similarly, the adjusted total hip BMD was higher in the highest UA quartile at 0.847 g/cm^2^ (95% CI: 0.840–0.854) compared with 0.828 g/cm^2^ (95% CI: 0.821–0.834) in the lowest quartile (*P*=0.001).

**Conclusion:**

Our results suggest that elevated serum UA levels are associated with higher BMD in peri- and postmenopausal Korean women, indicating a potential protective role in bone metabolism.

## Introduction

 Uric acid (UA) is the end-product of purine metabolism and has emerged as a molecule with complex roles in humans.^1,2^ It is recognized both as an antioxidant, which can reduce oxidative stress, and as a pro-oxidant, potentially contributing to inflammation and pathological processes such as endothelial dysfunction and insulin resistance.^3,4^ Oxidative stress is a significant factor in bone resorption, leading to conditions such as osteoporosis, particularly in post-menopausal women.^5^ Several studies have suggested that UA may influence bone metabolism through its interactions with oxidative stress pathways.^6^ As an antioxidant, UA may protect bone by reducing oxidative damage to osteoblasts and limiting osteoclast activation. Conversely, in certain conditions, UA may act as a pro-oxidant, promoting inflammation and contributing to bone resorption. The balance between these opposing effects may be influenced by factors such as underlying metabolic conditions, inflammation, and the local bone microenvironment.^6,7^

 Osteoporosis is a skeletal disorder characterized by reduced bone mass and deterioration of bone tissue, leading to increased fracture risk.^8^ Bone mineral density (BMD) is a key indicator of bone health, and its decline is well-established in postmenopausal women due to reduced estrogen levels.^9^ However, previous studies examining the relationship between serum UA levels and BMD have reported conflicting results.^10^ Some research indicates that higher UA levels are associated with higher BMD,^6,7^ while others suggest no association or even potential harm from elevated UA levels.^11,12^ These discrepancies may stem from differences in study design (cross-sectional vs. longitudinal), variations in population characteristics (age, sex, ethnicity, and comorbidities), and inconsistencies in measurement techniques (dual-energy X-ray absorptiometry vs. quantitative computed tomography).

 Given these inconsistencies, further exploration of the relationship between UA and BMD is necessary, particularly in populations at risk for osteoporosis, such as peri- and postmenopausal women. Considering the rising prevalence of osteoporosis and its significant impact on healthcare systems globally, clarifying the relationship between UA and BMD may provide essential insights for developing targeted public health strategies and preventive measures, thereby reducing fracture risks and improving quality of life

 Therefore, this study aimed to investigate the association between serum UA levels and BMD of the lumbar spine and hip in peri- and postmenopausal Korean women.

## Materials and Methods

###  Study Design and Participants

 This cross-sectional study included 5312 female participants who underwent BMD assessments as part of a voluntary general health examination between March 2014 and March 2020 at Ulsan University Hospital, a single tertiary medical center located in Ulsan, South Korea. These screenings were offered to individuals seeking preventive healthcare services primarily within the Ulsan metropolitan area. The study population predominantly comprised residents from this area. Participants were not actively recruited but were selected based on pre-existing health records from the clinical data warehouse platform of our institution.

 Among these patients, we excluded (*i*) those outside the age range of 50 to 80 years (n = 1611); (*ii*) participants with chronic kidney disease (estimated glomerular filtration rate [eGFR] ≤ 60 mL/min/1.73 m^2^) (n = 122); (*iii*) participants with hyperthyroidism (n = 2) or hypothyroidism (n = 8); and (*iv*) participants who did not have measured serum UA levels (n = 3). After applying the exclusion criteria, a total of 3566 women were included in the study.

###  Clinical and Laboratory Measurements

 Clinical and laboratory data were obtained from the electronic medical records and the clinical data warehouse platform of our institution. Height, body weight, and blood pressure were measured according to standard procedures during the general health examination.^13^ Blood samples taken after overnight fasting were analyzed for total cholesterol, fasting glucose, hemoglobin A1c, alkaline phosphatase, vitamin D, creatinine, UA, and C-reactive protein levels. Diabetes was defined as fasting plasma glucose ≥ 126 mg/dL, hemoglobin A1c ≥ 6.5%, or a self-reported history of diabetes or treatment with dietary modifications or antidiabetic medication. Laboratory assays were conducted in a certified clinical laboratory using automated analyzers, with internal and external quality control procedures in place to maintain consistency across all data collection points. Hypertension was defined as systolic blood pressure ≥ 140 mm Hg, diastolic blood pressure ≥ 90 mm Hg, or a self-reported history of hypertension or treatment with antihypertensive medication. Hyperlipidemia was defined as total cholesterol ≥ 240 mg/dL or a self-reported history of hyperlipidemia or treatment with anti-hyperlipidemic medications.^13^

###  Measurement of Bone Mineral Density

 BMD was assessed using dual-energy X-ray absorptiometry scans (Horizon®, HOLOGIC; USA), with routine daily calibration using a standard phantom provided by the manufacturer to ensure measurement accuracy. The coefficient of variation for BMD measurements at our institution was < 1.5%. Measurements were obtained at two primary sites: the lumbar spine (L1–L4) and the hip (including the femoral neck, trochanter, and intertrochanter regions).

###  Statistical Analysis

 Participants were stratified into quartiles according to their serum UA levels. Categorical variables are expressed as frequencies with percentages, while continuous variables are expressed as means and standard deviations. Between-group comparisons were performed using Pearson’s chi-square test for categorical variables, and one-way analysis of variance was employed for numerical variables, as the assumptions of normality and homogeneity of variance were met. Multivariate analysis was performed using analysis of covariance (ANCOVA) to evaluate the association between serum UA levels and BMD. The selection of covariates for the multivariate analysis was included by both clinical significance and statistical relevance. The covariates were adjusted gradually in three models. The initial model, Model 1, incorporated corrections for age and body mass index (BMI). Building upon this, Model 2 included additional adjustments for lifestyle and clinical factors, such as current smoking status, diabetes, and hypertension. The final model, Model 3, expanded the analysis further by integrating biochemical markers, including serum alkaline phosphatase, serum vitamin D, and eGFR. The normality of continuous variables was assessed using the Shapiro-Wilk test and histogram visualization. Levene’s test was performed to check for homogeneity of variance. Linearity was evaluated using scatter plots, and multi-collinearity among covariates in the multivariate model was assessed using variance inflation factor (VIF), with VIF < 10 considered acceptable. A post hoc power analysis was conducted to assess whether the sample size was sufficient to detect meaningful differences in BMD across UA quartiles. The analysis confirmed that the study had adequate power ( > 80%) to detect moderate effect sizes at a significance level of 0.05. All *P *values were two-sided, and *P* < 0.05 was considered statistically significant. Data manipulation and statistical analyses were performed using the SPSS software (version 24; SPSS Inc., Chicago, IL, USA).

## Results

 The mean age of the 3566 participants was 56.9 ± 5.8 years, and the mean serum UA level was 4.41 ± 0.97 mg/dL. The baseline characteristics of the study participants stratified by serum UA quartiles are shown in Table 1. BMI and the prevalence of hyperlipidemia increased significantly with higher serum UA quartiles. Additionally, serum alkaline phosphatase levels increased with higher serum UA levels, while GFR decreased.

**Table 1:** Baseline Characteristics of the Study Population According to the Quartiles of Serum Uric Acid Levels.

**Characteristics **	**Total** **(n=3566)**	**Serum Uric Acid**				***P*** ** Value**
		**Quartile 1** **≤3.80 mg/dL** **(n=867)**	**Quartile 2** **3.81–4.30 mg/dL** **(n=929)**	**Quartile 3** **4.31–5.10 mg/dL** **(n=945)**	**Quartile 4** **≥5.11 mg/dL** **(n=825)**	
Age, years	56.9 ± 5.8	57.0 ± 6.0	56.5 ± 5.8	56.8 ± 5.7	57.3 ± 5.6	0.030
Body mass index, kg/m^2^	23.6 ± 2.9	23.0 ± 2.7	23.2 ± 2.6	23.8 ± 3.0	24.6 ± 3.2	< 0.001
Current smoker	56 (1.6%)	12 (1.4%)	9 (1.0%)	12 (1.3%)	23 (2.8%)	0.012
Diabetes mellitus	250 (7.0%)	81 (9.3%)	52 (5.6%)	50 (5.3%)	67 (8.1%)	0.001
Hypertension	723 (20.3%)	177 (20.4%)	139 (15.0%)	174 (18.4%)	233 (28.2%)	< 0.001
Hyperlipidemia	206 (5.8%)	41 (4.7%)	45 (4.8%)	61 (6.5%)	59 (7.2%)	0.075
WBC, k/mmL	5.141 ± 1.45	4.993 ± 1.44	5.178 ± 1.45	5.023 ± 1.41	5.287 ± 1.44	0.097
Hb, g/dL	13.3 ± 1.0	13.4 ± 0.9	13.2 ± 1.0	13.1 ± 1.0	13.4 ± 0.9	0.312
Platelet, k/mmL	237.7 ± 53.4	235.4 ± 51.5	240.1 ± 52.8	236.8 ± 50.1	238.6 ± 58.6	0.156
Total protein, g/dL	7.0 ± 0.5	7.0 ± 0.4	6.9 ± 0.5	7.1 ± 0.5	7.0 ± 0.5	0.109
Total bilirubin, mg/dL	0.7 ± 0.3	0.7 ± 0.2	0.6 ± 0.2	0.7 ± 0.3	0.7 ± 0.3	0.267
ALP, IU/L	69.6 ± 21.4	67.7 ± 22.5	67.7 ± 19.6	69.9 ± 22.0	73.4 ± 21.2	< 0.001
Vitamin D, ng/mL	18.6 ± 9.7	19.0 ± 10.2	18.0 ± 9.5	18.1 ± 9.5	19.2 ± 10.1	0.124
C-reactive protein, mg/L	0.12 ± 0.5	0.12 ± 0.6	0.10 ± 0.4	0.12 ± 0.5	0.15 ± 0.4	0.067
GFR, mL/min/1.73 m^2^	90.9 ± 17.5	93.8 ± 17.6	90.5 ± 16.7	90.0 ± 18.0	89.1 ± 17.5	< 0.001

Values are shown as mean ± standard deviation or number (%). ALP: Alkaline phosphatase, GFR: Glomerular filtration rate.

 The associations between serum UA levels and BMD are listed in Table 2. At each lumbar spine site (L1, L2, L3, and L4), higher serum UA levels were consistently associated with greater BMD. The BMD of the total lumbar spine also exhibited a significant increase across quartiles of serum UA levels, rising from 0.892 g/cm^2^ in the first quartile to 0.918 g/cm^2^ in the fourth quartile. In the hip region, BMD at the femoral neck, trochanter, and total hip showed positive associations with serum UA levels.

**Table 2:** Bone Mineral Density According to the Quartiles of Serum Uric Acid Levels

**Site, BMD (g/cm**^2^**)**	**Total** **(n=3566)**	**Serum Uric Acid**				***P*** ** Value**
		**Quartile 1** **≤3.80 mg/dL** **(n=867)**	**Quartile 2** **3.81–4.30 mg/dL** **(n=929)**	**Quartile 3** **4.31–5.10 mg/dL** **(n=945)**	**Quartile 4** **≥5.11 mg/dL** **(n=825)**	
L1 spine	0.823 ± 0.129	0.814 ± 0.129	0.818 ± 0.131	0.827 ± 0.128	0.833 ± 0.126	< 0.001
L2 spine	0.879 ± 0.141	0.867 ± 0.140	0.873 ± 0.143	0.884 ± 0.140	0.894 ± 0.140	< 0.001
L3 spine	0.932 ± 0.151	0.914 ± 0.147	0.924 ± 0.153	0.941 ± 0.151	0.950 ± 0.149	< 0.001
L4 spine	0.959 ± 0.161	0.941 ± 0.157	0.947 ± 0.160	0.965 ± 0.159	0.985 ± 0.164	< 0.001
L spine, total	0.903 ± 0.140	0.888 ± 0.138	0.895 ± 0.141	0.909 ± 0.139	0.921 ± 0.139	< 0.001
Femur neck	0.689 ± 0.104	0.682 ± 0.106	0.685 ± 0.103	0.692 ± 0.103	0.700 ± 0.101	0.002
Trochanter	0.611 ± 0.092	0.600 ± 0.093	0.607 ± 0.089	0.615 ± 0.092	0.624 ± 0.092	< 0.001
Hip, total	0.836 ± 0.113	0.822 ± 0.114	0.832 ± 0.111	0.840 ± 0.114	0.852 ± 0.110	< 0.001

Values are shown as mean ± standard deviation. BMD: Bone mineral density, L: Lumbar.

 In the multivariate analysis, a significant association was observed between serum UA levels and BMD across all measured sites. This association remained significant in Model 1, which adjusted for age and BMI, and persisted in Model 2, which further accounted for lifestyle and clinical factors, including current smoking status, diabetes, and hypertension. In Model 3, which incorporated biochemical markers such as serum alkaline phosphatase, serum vitamin D, and eGFR, the association was still maintained, indicating that the relationship between serum UA and BMD is independent of these additional adjustments (Table 3, Supplementary file 1, Tables S1 and S2). Bonferroni post hoc comparisons between the quartiles indicated significant differences, with participants in the fourth UA quartile consistently showing higher BMD than those in the first or second quartile (Table 3).

**Table 3:** Adjusted Mean Value of Bone Mineral Density According to the Quartiles of Serum Uric Acid Levels

**Site, BMD (g/cm**^2^**)**	**Serum Uric Acid**				***P*** Value^*^
	**Quartile 1** **≤3.80 mg/dL** **(n=867)**	**Quartile 2** **3.81–4.30 mg/dL** **(n=929)**	**Quartile 3** **4.31–5.10 mg/dL** **(n=945)**	**Quartile 4** **≥5.11 mg/dL** **(n=825)**	
L1 Spine	0.816 (0.808–0.823)^a^	0.816 (0.809–0.823)^b^	0.826 (0.818–0.833)	0.835 (0.827–0.843)^a,b^	0.001
L2 Spine	0.870 (0.862–0.878)^a^	0.871 (0.863–0.880)^b^	0.883 (0.875–0.891)	0.893 (0.884–0.902)^a,b^	< 0.001
L3 Spine	0.918 (0.909–0.927)^a,b^	0.923 (0.914–0.932)^c^	0.939 (0.930–0.948)^a^	0.947 (0.938–0.956)^b,c^	< 0.001
L4 Spine	0.946 (0.936–0.956) ^a^	0.948 (0.939–0.958)^b^	0.962 (0.953–0.972)	0.979 (0.969–0.989)^a,b^	< 0.001
L Spine, Total	0.892 (0.884–0.900) ^a^	0.894 (0.886–0.902) ^b^	0.907 (0.899–0.915)	0.918 (0.909–0.927)^a,b^	< 0.001
Femoral Neck	0.686 (0.679–0.692)	0.685 (0.679–0.691) ^a^	0.690 (0.684–0.696)	0.697 (0.691–0.704) ^a^	0.026
Trochanter	0.605 (0.600–0.611) ^a^	0.609 (0.604–0.614)	0.613 (0.608–0.618)	0.618 (0.613–0.624) ^a^	0.008
Hip, Total	0.828 (0.821–0.834)^a^	0.833 (0.827–0.840)^b^	0.838 (0.832–0.844)	0.847 (0.840–0.854)^a,b^	0.001

BMD: Bone mineral density, L: Lumbar. Variables are expressed as means (95% confidence interval). Covariates in the multivariate analysis included age, body mass index, current smoking status, diabetes, hypertension, serum alkaline phosphatase, serum vitamin D, and glomerular filtration rate. ^*^P value was obtained by analysis of covariance. ^a,b,c^ Superscript letters indicate statistically significant differences between quartiles within the same row, based on Bonferroni post hoc analysis.

## Discussion

 In this study, we investigated the relationship between serum UA levels and BMD in a cohort of peri- and postmenopausal Korean women. Our findings demonstrated a positive association between higher serum UA levels and increased BMD in both the lumbar spine and hip regions, indicating that higher UA levels may be linked to better bone health in peri- and postmenopausal women

 Oxidative stress is a major factor in the pathogenesis of osteoporosis; this process accelerates bone resorption by promoting osteoclast activity and decreasing osteoblast function.^14^ UA, which can act as an antioxidant, may counteract oxidative stress and damage through several mechanisms. Firstly, UA neutralizes various oxidants, such as superoxide anions, hydrogen radicals, and particularly peroxynitrite, a potent oxidant that can trigger inflammatory responses, lipid peroxidation, and tyrosine nitration.^15^ Peroxynitrite is formed when nitric oxide (NO) combines with superoxide, leading to oxidative stress. However, UA helps mitigate these effects by reducing superoxide levels and protecting NO production by preventing the uncoupling of NO synthase.^16^ Secondly, UA is a highly effective scavenger for peroxyl radicals (ROO^-^) and outperforms ascorbic acid as the primary water-soluble antioxidant in humans due to its higher concentration and reduction potential.^17^ Additionally, by limiting the production of iron and copper ions, UA reduces hydroxyl radical generation via the Fenton reaction and acts as an iron chelator, minimizing iron-catalyzed oxidative stress.^18,19^ Lastly, UA can attenuate the formation of nitrotyrosine (a footprint of peroxynitrite) in injured tissue and reduce neutrophil infiltration, preventing oxidative stress-induced inflammation.^20^ Therefore, UA’s antioxidant properties may protect bones by mitigating the harmful effects of oxidative stress.

 However, UA may also have detrimental effects on bone health. In hyperuricemia or gout, the intracellular degradation of UA generates reactive oxygen species, which could lead to increased oxidative stress within bone cells.^3^ This stress may stimulate osteoclast activity, promote bone resorption, and trigger the release of inflammatory cytokines, potentially exacerbating bone loss. A study by Lin et al^21^ described that chronically elevated UA levels are associated with increased bone turnover and reduced bone quality due to the enhanced activity of pro-inflammatory cytokines. Moreover, high UA levels impair vitamin D metabolism by inhibiting the urate transporter, ATP-binding cassette subfamily G member 2, leading to secondary hyperparathyroidism, a condition known to stimulate bone resorption and accelerate bone loss.^22^

 Previous clinical studies have reported conflicting results regarding the association between serum UA levels and BMD, reflecting the complexity of UA’s role in bone metabolism. While some studies suggest that UA exerts a protective effect due to its antioxidant properties, others have reported no significant association or even detrimental effects, particularly at high concentrations. Makovey et al^23^ and Ahn et al^24^ demonstrated a positive relationship between UA and BMD, while Kang et al^11^ found no significant association among postmenopausal Korean women. Conversely, studies by Paik et al^25^ and Ibrahim et al^7^ suggest that excessive UA levels may contribute to increased fracture risk and reduced bone quality, potentially mediated by chronic inflammation and impaired vitamin D metabolism. These discrepancies underscore the need to consider potential confounding factors such as metabolic status, renal function, and systemic inflammation, which may modify UA’s effects on bone health. From a broader public health perspective, these findings highlight the importance of identifying individuals who may benefit from maintaining moderate UA levels while mitigating risks associated with hyperuricemia. Future research should aim to define clinical thresholds at which UA transitions from a protective factor to a potential risk factor for bone loss, ultimately guiding more personalized approaches to osteoporosis prevention.

 While this study identified a statistically significant correlation between elevated serum UA levels and increased BMD, it is crucial to assess the clinical relevance of this finding. Although the observed effect size was substantial, further analysis is necessary to ascertain its actual impact on bone health in clinical settings. Statistical significance does not inherently imply clinical significance, and the magnitude of the effect, though notable, may not be sufficient to justify a significant alteration in clinical practice without additional validation. Specifically, the positive association between serum UA and BMD was modest, indicating that while UA may contribute to bone health, it should not be considered the sole determinant of BMD or a standalone intervention for osteoporosis prevention. The intricate relationship between serum UA levels and bone metabolism, along with the potential for both beneficial and adverse effects at varying UA levels, highlights the need for careful interpretation of these findings. Clinicians should evaluate the broader context, including individual risk factors for osteoporosis, when considering the potential role of UA in bone health.

 This study has several strengths. It analyzed a relatively large sample of peri- and postmenopausal women, allowing for a more robust evaluation of the relationship between serum UA levels and BMD. Furthermore, we adjusted for multiple confounding factors, including BMI, lifestyle factors, and metabolic parameters, strengthening the validity of our findings. Additionally, the use of standardized BMD measurements and a well-defined study population enhances the reproducibility of our results. However, several limitations should be considered. First, it was a single-center study, and all participants voluntarily visited the hospital for general health screening, introducing potential selection bias and confounding effects. Second, this study focused exclusively on post-menopausal Korean women, which may limit the applicability of the results to other populations and demographics. Lastly, this study did not collect data on hormone replacement therapy status, menopause age, or reproductive history, which could be important confounders in the association between serum UA and BMD. Therefore, further research with larger sample sizes and diverse populations, and more comprehensive confounding data is necessary to better understand the dual effects of UA on bone metabolism and to generalize the findings across different ethnicities.

 Clinicians may consider monitoring serum UA levels as part of routine health screenings for peri- and postmenopausal women, especially those at higher risk for osteoporosis. Early identification of elevated UA levels could prompt further evaluation of bone health and facilitate early intervention. In at-risk populations, maintaining optimal UA levels may be an important factor in preventing further bone deterioration. Personalized approaches to osteoporosis prevention, based on a comprehensive evaluation of individual risk factors, could further optimize clinical management.

## Conclusion

 Our study demonstrated that higher serum UA levels were associated with increased BMD in the lumbar spine and hip in peri- and postmenopausal Korean women. While the antioxidant properties of UA may explain this association, further research is needed to confirm these findings and elucidate the paradoxical mechanism by which UA influences bone health. Balancing UA levels and monitoring their effects on bone health may be crucial for developing effective strategies to prevent and manage osteoporosis in peri- and postmenopausal women. However, given the cross-sectional design of our study, more longitudinal and prospective research is needed before considering UA as a therapeutic target for osteoporosis prevention. Cautious interpretation is warranted, and clinical recommendations should be based on further evidence from more diverse populations and extended follow-up studies.

## Supplementary Files

Supplementary file 1 contains Tables S1 and S2 (Adjusted mean value of bone mineral density).
