# Supplementary material for: Serum Uric Acid Levels and Bone Mineral Density in Peri- and Postmenopausal Korean Women: A Cross-sectional Study on 3,566 Cases
Source: Arch Iran Med. 2025 Jun 1;28(6):341–6. doi: 10.34172/aim.34066 (PMC12305421; doi:10.34172/aim.34066)
Supplement: Supplementary file 1 — contains Tables S1 and S2 (Adjusted mean value of bone mineral density). [file aim-28-341-s001.pdf]

Arch Iran Med. June 2025;28(6):341-S1
10.34172/aim.34066

table S1. Adjusted Mean Value of Bone Mineral Density According to the Quartiles of Serum Uric Acid Levels (Adjusted for age and body mass index).

Site, BMD (g/cm^2)	Serum Uric Acid				
	Quartile 1<br>≤ 3.80 mg/dL<br>(n = 867)	Quartile 2<br>3.81–4.30 mg/dL<br>(n = 929)	Quartile 3<br>4.31–5.10 mg/dL<br>(n = 945)	Quartile 4<br>≥ 5.11 mg/dL<br>(n = 825)	*P* value*
L1 Spine	0.819 (0.811-0.827) [a]	0.818 (0.810-0.825) [b]	0.825 (0.817-0.832)	0.835 (0.827-0.843) [a, b]	0.017
L2 Spine	0.873 (0.864-0.881) [a]	0.874 (0.866-0.883)	0.883 (0.874-0.891)	0.891 (0.882-0.899) [a]	0.011
L3 Spine	0.921 (0.912-0.930) [a, b]	0.926 (0.917-0.935)	0.939 (0.930-0.948) [a]	0.941 (0.932-0.951) [b]	0.005
L4 Spine	0.949 (0.939-0.959) [a]	0.951 (0.941-0.961) [b]	0.962 (0.953-0.972)	0.973 (0.963-0.984) [a, b]	0.004
L Spine, Total	0.895 (0.886-0.903) [a]	0.897 (0.888-0.905)	0.907 (0.899-0.915)	0.913 (0.904-0.921) [a]	0.001
Femoral Neck	0.687 (0.681-0.694) [a]	0.686 (0.680-0.692) [b]	0.690 (0.684-0.696)	0.694 (0.688-0.701) [a, b]	0.026
Trochanter	0.607 (0.602-0.613) [a]	0.610 (0.605-0.616)	0.613 (0.607-0.618)	0.619 (0.614-0.625) [a]	0.018
Hip, Total	0.830 (0.823-0.836) [a]	0.835 (0.828-0.841)	0.838 (0.831-0.844)	0.843 (0.836-0.850) [a]	0.013

Variables are expressed as means (95% confidence interval).
*P value was obtained by analysis of covariance.
[a,b] Superscript letters indicate statistically significant differences between quartiles within the same row, based on Bonferroni *post-hoc* analysis.

table S2. Adjusted Mean Value of Bone Mineral Density According to the Quartiles of Serum Uric Acid Levels (Adjusted for age, body mass index, current smoking status, diabetes, and hypertension).

Site, BMD (g/cm^2)	Serum Uric Acid				
	Quartile 1<br>≤ 3.80 mg/dL<br>(n = 867)	Quartile 2<br>3.81–4.30 mg/dL<br>(n = 929)	Quartile 3<br>4.31–5.10 mg/dL<br>(n = 945)	Quartile 4<br>≥ 5.11 mg/dL<br>(n = 825)	*P* value*
L1 Spine	0.818 (0.810-0.826)	0.818 (0.811-0.826)	0.825 (0.818-0.833)	0.830 (0.822-0.838)	0.001
L2 Spine	0.873 (0.864-0.881)	0.874 (0.866-0.882)	0.883 (0.874-0.891)	0.888 (0.879-0.897)	< 0.001
L3 Spine	0.921 (0.912-0.930) [a, b]	0.926 (0.917-0.935)	0.939 (0.930-0.948) [a]	0.941 (0.932-0.951) [b]	0.005
L4 Spine	0.949 (0.939-0.959) [a]	0.951 (0.941-0.961) [b]	0.962 (0.953-0.972)	0.973 (0.963-0.984) [a, b]	0.004
L Spine, Total	0.895 (0.886-0.903) [a]	0.897 (0.888-0.905)	0.907 (0.899-0.915)	0.913 (0.904-0.921) [a]	0.014
Femoral Neck	0.687 (0.681-0.694)	0.686 (0.680-0.692) [a]	0.690 (0.684-0.696)	0.697 (0.693-0.704) [a]	0.024
Trochanter	0.607(0.602-0.613) [a]	0.610(0.605-0.616)	0.613(0.607-0.618)	0.619 (0.614-0.625) [a]	0.012
Hip, Total	0.830 (0.823-0.836) [a]	0.835 (0.828-0.841)	0.838 (0.831-0.844)	0.843 (0.836-0.850) [a]	0.007

Variables are expressed as means (95% confidence interval).

**P* value was obtained by analysis of covariance.

[a,b] Superscript letters indicate statistically significant differences between quartiles within the same row, based on Bonferroni *post-hoc* analysis.
